# Stone Milling versus Roller Milling in Soft Wheat: Influence on Products Composition

**DOI:** 10.3390/foods9010003

**Published:** 2019-12-19

**Authors:** Marina Carcea, Valeria Turfani, Valentina Narducci, Sahara Melloni, Vincenzo Galli, Valentina Tullio

**Affiliations:** Research Centre for Food and Nutrition, Council for Agricultural Research and Economics (CREA), Via Ardeatina 546, 00178 Rome, Italyvalentina.narducci@crea.gov.it (V.N.); sahara.melloni@crea.gov.it (S.M.); vincenzo.galli@crea.gov.it (V.G.); valentinatullio.nu@gmail.com (V.T.)

**Keywords:** soft wheat, wholegrain flour, stone milling, roller milling, composition, lipids, dietary fibre, polyphenols, alkylresorcinols

## Abstract

Wholegrain wheat flours are in great demand from consumers worldwide because they are considered healthier then refined flours. They can be obtained by either stone milling, which is experiencing a revival in Europe, or roller milling. In order to study compositional differences due to the milling technology and to explore the possibility of a better qualification of wholegrain flours by means of nutritionally oriented quality parameters, eight mixes of soft wheat grains were stone milled and roller milled and the milling products were analyzed for their protein, ash, lipids, total dietary fibre, total polyphenols and alkylresorcinols content. A wholegrain flour milled with a laboratory disk mill was used as a comparison and a set of seven wholegrain flours purchased on themarket were also analyzed and compared. The particle size distribution of stone milled and recombined roller milled flour was also studied. Considering the above mentioned parameters, we found that there is no compositional difference between a stone milled or a roller milled flour if, in this latter one, the milling streams are all recombined, but the particle size distribution was different. This might have an impact on the technological quality of flours and on the bioavailability of components.

## 1. Introduction

It is now recognized worldwide that a human diet rich in wholegrains reduces a number of health risks including cardiovascular diseases, colorectal cancer, type 2 diabetes, overweight and obesity [1] and several governments and health promoting organizations recommend more consumption of wholegrain food. This has increased demand from consumers and processing industry for cereal products that are wholegrain, wheat wholegrain flours in particular.

Wheat kernels consist of three main parts: endosperm, bran and germ from the center to the external zone. The outer layers making up the bran and germ are richer in dietary fibre and bioactive components including lipids which are therefore unevenly distributed in the grain. The milling processes have an impact on the presence of all the kernel components in the flour therefore on its nutritional quality, but also on the flour particle size which determines the flour technological and nutritional functionality [2].

The two predominant techniques for grinding whole grain flours are stone milling and roller milling. Whole grain flours could also notionally be produced with an impact or hammer mill, but this is rarely used [3].

Stone mills are the oldest attrition mills used for making whole grain flours, which simultaneously use compression, shear, and abrasion to grind wheat kernels between two stones and produce a theoretical extraction rate of 100% [4]. Modern stone mills are metal plates with composition stones attached [5]. Stone mills generate considerable heat due to friction. This can result in considerable damage to starch, protein, and unsaturated fatty acids in comparison with other milling techniques [6]. The production of flour by stone milling has recently witnessed a revival because consumers associate it with an idea of integrity. Sometimes stone milled flours are also sifted to comply with current legislation regarding wholegrain flour composition in the different countries.

The process of roller milling involves separation of the endosperm from the bran and germ followed by gradual size reduction of endosperm [7]. In this process, wheat is passed through a series of corrugated and smooth metal rollers accompanied by sifting between stages. Producing flour that fulfils the requirement for being whole grain is achieved by blending bran and germ back with the endosperm flour, possibly in the naturally-occurring proportions.

A definition of wholegrain and wholegrain product is required as a basis for dietary recommendations, labelling, legislation, health claims, nutrition research to know exactly which materials are studied, and some attempts have recently been made by scientific international organizations such as the American Association of Cereal Chemists International (AACCI) and the Healthgrain Forum to internationally agree on definitions which, taking into account current technological practices, could clearly list the specifications for a flour or a food product to be identified as wholegrain [8,9].

However, definitions in order to be useful should be substantiated and supported by compositional data and quality parameters which allow to clearly identify and distinguish wholegrain products from non-wholegrain products but also different wholegrain products, in the interest of industry and consumers. For example, in Italy the current legislation for wheat flours (both soft and durum wheat) makes a distinction between wholegrain flour and refined flours based on a minimum and maximum value of ash content and a minimum value of protein content only.

If a more nutritionally oriented legislation that promotes the production of wholegrain products has to be implemented, maybe some new quality parameters more related to the bioactive components of wholegrain flours could be considered. Within the EU FP6 HEALTHGRAIN project a wheat germplasm collection of 150 genotypes was analyzed for a range of phytochemicals (tocols, sterols, phenolic acids, pholates, alkylresorcinols, dietary fibre components) showing that significant variation was present in all groups of components and that grain composition is affected by genetics, environment and agronomy [10,11].

With this idea in mind, we set out to measure some additional quality parameters besides protein and ash that based on existing literature could be quantified in a reproducible manner and could describe the nutritional value of wholegrain soft wheat flours. We selected total lipids, free and bound polyphenols, soluble and insoluble dietary fibres and alkylresorcinols. We chose to analyze samples coming from the same grains milled in two different ways, namely stone milling and roller milling, to assess differences in the same parameters due to processing. A set of wholemeal flours bought on the market were also analyzed as comparison.

Our paper will contribute compositional data to the current knowledge on the nutritional quality of wholegrain soft wheat flours and they will be useful for composition tables, legislators, nutritional studies, food industry and consumers.

## 2. Materials and Methods

### 2.1. Sampling, Milling and Sample Preparation for Analysis

Eight samples of stone milled soft wheat flours (SMF) produced by different mills in Central Italy, plus their corresponding clean grains (*Triticum aestivum* L.) before milling, were collected at the manufacturer. The grains were a mix of different varieties, coming from different locations and they represented the raw material commonly used by commercial mills.

The same grains obtained from the commercial stone mills were roller milled (see below) in our laboratory to obtain 3 different mill streams, namely refined white flour (RF), coarse bran (CB) and fine bran (FB). The grains were also ground by means of a laboratory disc mill (see below) to produce a whole grain flour (WGF) which was used as reference of a perfect wholemeal flour. So starting from the 8 grain samples, 40 flour samples were obtained.

In addition to the above samples, 7 commercial whole wheat flours from 6 different brands were purchased in stores. Three of them were labelled as stone milled wholemeal, the other 4 as wholemeal only, surely roller milled. These commercial flours (C) were used as comparison.

For roller milling grains were tempered to 15.5–17.5% moisture (for 36–48 h depending on their hardness measured by means of the SKCS 4100 instrument, Perten Instruments, Stockholm, Sweden) and subsequently milled in a Bühler MLU 202 pilot mill (Bühler, Uzwill, Switzerland) equipped with three break rolls, three reduction rolls and six screens, according to method 26-10.02 of the AACCI [12] with an average of 71% extraction rate for the refined flour. Roller milling of wheat produces various fractions with different physicochemical characteristics: F1 (refined flour), F2 (fine bran) and F3 (coarse bran). The germ is found mixed with the bran fractions.

Grains were also ground in a Bühler MLI 204 laboratory disc mill to obtain wholegrain flour (WGF) with ≤0.5 mm particles.

Particle size distribution of flours after stone and roller milling was determined by a mechanical sifter (Bühler MLI 300 B, Bühler, Uzwill, Switzerland) by using 100 g of flour and 5 min sifting time. The sifter was equipped with the following 6 sieves: 38GG (494 µm), 48GG (363 µm), 8xx (183 µm), 10xx (129 µm), 15xx (85 µm), 25PR (35 µm).

Sample preparation before analyses implied that all samples were sifted before analyses and the residue not passing the 494 μm sieve was ground again by the Bühler MLI 204 laboratory mill until it all passed through the sieve.

### 2.2. Chemicals

Methanol, 96% (*w*/*w*), ethyl alcohol (96% *w*/*w*), n-hexane, ethyl acetate, formic acid (99% *w*/*w*) hydrochloric acid (37% *w*/*w*) and anhydrous sodium sulphate were of analytical grade and were purchased from Carlo Erba (Milan, Italy). Folin-Ciocalteu reagent and sodium carbonate 20% (*w*/*w*) solution were purchased from Carlo Erba (Milan, Italy). Gallic acid, n-5-pentadecylresorcinol and Fast Blue B Zn salt (CAS Number 14263-94-6) were purchased from Sigma (Saint Louis, MO, USA). Acetone was from VWR International (Radnor, PA, USA). All solvents were of analytical grade and reagents were of the highest available purity.

### 2.3. Analyses

Test weight of the grains was determined by means of a Shoppers condrometer and grain moisture by means of the Aquasearch P600 instrument (Kett Electric Laboratory, Tokio, Japan).

The 40 milling products (stone-milled flours, roller-milled fractions and wholegrain flours) were analyzed for proximate composition, polyphenols and alkylresorcinols according to the methods described below. The 7 flours purchased on the market were analyzed for the parameters not reported on the label only, i.e., moisture, ash, polyphenols, and alkylresorcinols.

Moisture was determined by oven drying to constant weight according to ICC Method N. 110/1 [13]; total protein by the Kjeldahl method according to AOAC Official Method 2001.11 [14] using 5.70 as a conversion factor, ash in a muffle furnace according to ICC Method N. 105/2 [13], total fat by hydrolysis in formic acid and hydrochloric acid at 75 °C reflux for 20 min followed by extraction in hexane and evaporation according to ICC Method N. 136 [13], and total dietary fibre (TDF) according to Lee et al. [15] using a reagent kit (K-TDFR, Megazyme Int., Wicklow, Ireland).

For total polyphenols determination, aqueous-organic extracts (extractable polyphenols) and their residues (non-extractable polyphenols) were isolated as described by Carcea et al. [16]. Two grams of sample (in triplicate) were placed into a Pyrex centrifuge tube (Vetroscientifica, Rome, Italy) with 20 mL of methanol/water 50:50 (*v*/*v*) equipped with a magnetic bar. The tube was left stirring at room temperature for 1 h, then it was centrifuged at 2500× *g* for 10 min and the supernatant was recovered. Twenty mL of acetone/water 70:30 (*v*/*v*) were added to the residue in the tube, the extraction and centrifugation steps were repeated, and the supernatants were combined. The resulting aqueous-organic extract was assayed for total phenolic content (TPC) as described below, whereas the wet extraction residue was left overnight in a ventilated oven at 30 °C. The dried residue was reduced to powder by a water-refrigerated laboratory mill and its residual moisture was checked (according to the abovementioned standard method). Hence, the powdered dried residue was hydrolysed in boiling methanol/sulfuric acid according to Hartzfeld et al. [17]. Two-hundred milligrams of powdered dried residue were placed in a Pyrex tube (200 mm high, 250 mm wide) equipped with a magnetic bar and screw cap. Twenty mL of methanol and 2 mL of concentrated sulfuric acid were added and the tube was placed in an aluminum block over a magnetic hot plate equipped with a Vertex thermoregulator (Velp, Usmate Velate, Italy) under stirring for 16 h. After this time, the tube content was transferred into a graduated cylinder (50 mL). The cylinder was cooled in an ice bath and the pH was adjusted between 2 and 3 under stirring by dropwise addition of 8 M NaOH. An aliquot of the mixture was transferred into a Pyrex centrifuge tube and centrifuged at 2500× *g* for 10 min. The supernatant, containing hydrolysable bound polyphenols, was recovered and assayed for TPC. The TPC assay was carried out by means of the Folin-Ciocalteau reagent as described by Singleton et al. [18], absorbance was measured at 760 nm against a blank after 2 h of reaction at room temperature and gallic acid was used as a standard. Mean values of triplicate determinations plus standard deviation (SD) and ranking are reported. The sum of TPC of both fractions was expressed as total polyphenols.

Alkylresorcinols were determined by a rapid colorimetric method [19]. One gram of flour sample (0.3 g for bran fractions) were placed into 50-mL tubes and extracted with 40 mL of acetone under magnetic stirring for 48 h at room temperature [20]. The extracts were filtered through a medium-flow filter paper (Whatman Grade 1, 11 μm particle retention) and evaporated to dryness by a rotary evaporator connected to a Laboport vacuum pump (KNF, Neuberger, Germany). For the colorimetric assay, the dry residues were redissolved in 1 mL ethyl acetate and 10 μL were transferred in a vial, then the solvent was evaporated under a nitrogen stream, 2 mL of fresh diazo reagent (Fast Blue B Zn) were added and after mixing the sample was stored in a dark place away from light. After 60 min of incubation, the absorbance was read at 520 nm wavelength (1 cm optical path) against a reagent blank. The total ARs content in the sample was estimated by means of the appropriate calibration curve prepared by using n-5-pentadecylresorcinol from wheat as reference compound (from 0.1 to 7 mg of standard assayed by the Fast Blue B Zn procedure).

### 2.4. Data Presentation and Statistics

Total lipids and total dietary fibre were determined in duplicate. Protein, ash, polyphenols and alkylresorcinols were determined in triplicate. Means of all determinations accompanied by the Variation Coefficient (CV) are reported. All parameters except test weight, extraction rate and moisture are reported on a dry matter basis (d.m.).

One-way analysis of variance (factor = milling fraction) followed by Tukey’s honestly significant difference (HSD) test was performed by means of StatSoft Statistica 8.0 software (TIBCO Software Inc., Palo Alto, CA, USA). Other calculations were performed by Microsoft Excel.

## 3. Results

### 3.1. Proximate Composition of Flour Samples

The test weight of the eight grain samples and proximate composition of the 40 samples obtained from stone, roller milling and disc milling are presented in Table 1 together with the flour extraction rate.

The eight grain samples clearly represented different mixes of grains as can be seen from the test weight that went from 70.3 to 84.0 kg/hL. They also had different kernel hardness (grain mixes n. 1, 2 and 3 were medium, whereas all the others were hard), required different conditioning for roller milling (see Materials and methods section) and with this milling technique they gave milling streams with different extraction rates (from 62.4% to 78.7% for the refined flour, from 10.7% to 12.9% for coarse bran and from 10.5% to 13.8% for fine bran).

The grain samples had the same protein content as the wholemeal flour WGF i.e., the flour obtained with the Bühler MLI 204 laboratory mill and values went from 12.6% to 14.1% (d.m.). The same applies to the ash, lipids and total dietary fibre contents. This laboratory mill has no sieves and if correctly used reduces heat during milling, so that it is commonly used to guarantee the production of wholemeal flours in which kernel moisture, protein, and ash are preserved for analysis with standard methods. For the same reason, it is a good solution to preserve heat-sensitive compounds. The flours produced by this mill served in this study as a reference to evaluate whether the composition of stone-milled flours from commercial mills and wholegrain flours from the market was compatible with the presence of substantially all kernel components or not.

Total protein values (16.8–18.5% d.m. in CB, 15.7–16.8% in FB, 12.6–14.1% in WGF and SMF, 11.3–13.1% in RF) differed less between the five milled samples than the other parameters. In fact, roughly 80% of kernel protein is contained in the endosperm and the presence of the outer layers in the flour adds a 20% more only. However, it is known that proteins contained in the outer layers of wheat kernels (especially in the aleurone) have a nutritional importance since they are richer in essential amino acids than the endosperm proteins.

Coarse bran (CB) had ash in the range 6.03–7.23% d.m., fine bran (FB) had 3.38–4.15% d.m., wholegrain flour (WGF) and stone-milled flour (SMF) had 1.76–2.41% d.m. and refined flour (RF) had 0.54–0.98% d.m. only. These values reflect the presence of minerals in the outer layers of the wheat kernel.

The range of lipids in the grains was between 1.8% and 3.0% d.m. and the total lipid values in milled samples (6.1–7.5% d.m. in CB, 3.9–6.9% in FB, 1.8–3.0% in WGF and SMF, 0.7–1.7% in RF) are justified by the fact that wheat germ during roller milling is ground and ends up mainly in the two bran fractions, slightly more in the coarse than in the fine one. In wheat, lipids form 1–2% of the endosperm, 8–15% of the germ and about 6% of the bran, averaging 2–4% of the whole kernel [21].

Total dietary fibre values in grains ranged between 9.8% and 11.9% (d.m.). Total dietary fibre values in milled samples (39.8–46.5% d.m. in CB, 20.7–27.9% in FB, 10.2–11.9% in WGF and SMF, 1.8–2.7% in RF) show that fibre is contained almost exclusively in the outer kernel layers with coarse bran having the highest value thanks to presence of the outermost layers which are rich in insoluble dietary fibre.

The proximate composition of the seven commercial flours purchased in stores is illustrated in Table 2.

Protein contents of wholegrain samples purchased on the market ranged between 12.0–13.0% d.m., lipids between 1.8–1.9% d.m., ash between 1.55% and 1.79% d.m. and total dietary fibre between 7.6% and 12.5% d.m. If we compare these values with those in Table 1 we can notice that all the parameters are, on average, lower.

### 3.2. Total Polyphenols and Alkylresorcinols

The total polyphenol (TPF) and alkylresorcinol (AR) content of the 40 samples obtained by milling the eight mixes of grains is illustrated in Table 3.

Total polyphenols (TPF), expressed in mg of Gallic acid equivalent (GAE) per 100 g sample on d.m., ranged between 638 and 814 in grains, 1187–1547 in CB, 639–1126 in FB, 638–814 in WGF/SMF, 475–581 in RF. ANOVA showed clear significant differences (*p <* 0.05) between milling fractions as it also happened for proximate composition. Bran fractions were the richest in polyphenols (especially coarse bran), although refined flour was not devoid of them. The WGF and SMF resulted once more to be equal within Tukey’s HSD. A similar distribution was found for free and hydrolysable bound PF. Free PF represented 18–25% of total polyphenols for CB and FB, 31–35% of the total for RF and 25–31% of the total for WGF and SMF. Correspondingly, hydrolysable bound PF represented 66–82% of total PF for CB and FB, 65–71% of the total for RF and 69–75% for WGF/SMF.

Total alkylresorcinols, expressed in mg/100 g d.m., were 32.1–47.5 in grains, 168.4–251.2 in CB, 68.1–106.6 in FB, 32.1–47.5 in WGF/SMF, whereas they were below the limit of detection (l.o.d.) in RF. Again, ANOVA showed significant differences (*p <* 0.05) between milling fractions.

The polyphenol (PF) and alkylresorcinol (AR) content of the 7 commercial flours purchased in stores is illustrated in Table 4. Their total PF (597–678 mgGAE/100 g d.m.) and AR (27.9–38.1 mg/100 g d.m.) content was similar to that of WGF and SMF from the grain mixes, although towards the lowest limit of their range. This could be related to the varietal composition of the original grains from which these flours were milled but also to different processing conditions (sieving) after milling. No differences were found between stone-milled and not-stone-milled flours of this group.

### 3.3. Particle Size Distribution

**Figure 1:**
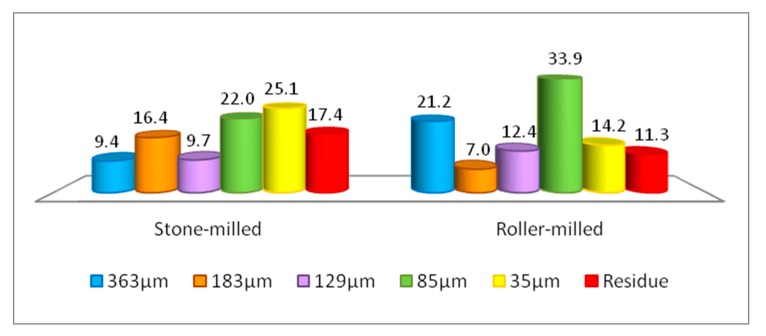
Average particle size distribution in wholegrain soft wheat flours obtained by stone milling and by recombining roller milling fractions (%); Values are averaged on eight samples.

The average particle size distribution in both whole meal stone milled flour and roller milled flour from the same grains is reported in Figure 1 where data coming from the 8 grains have been summed up and averaged in the graph. For the purpose of this measurement the streams of the roller mills have been reunited to produce a wholemeal flour as might happen in practice. It is evident from this figure that the distribution of flour particles is very different in the two kinds of flour: in roller milling the particles >363 µm and the particles between 129 µm and 85 µm represent 55% whereas in stone milling they are 31%. Moreover, in stone milling, there is a higher percentage of particles with a smaller size (>85 µm).

## 4. Discussion

The grain samples we studied could be considered representative of the raw materials that can be found in commercial milling, so we can say that our findings can have a general value.

Analysis of Variance (ANOVA) showed that total protein, ash, lipids and dietary fibre varied significantly (*p >* 5%) between the five different milling products, irrespectively of the grain mix of origin, reflecting the presence of the outer layers of the kernel (Table 1). Coarse bran had the highest values for all parameters, closely followed by fine bran, whereas refined flours had the lowest values. Wholegrain flour and stone milled flour had intermediate values, and for each grain, they resulted equal within the limits of Tukey’s Honestly Significant Difference (HSD).

It is noteworthy that all wholegrain flours (WGF and SMF from the eight pools) had ash above 1.70% d.m., representing the limit set by the Italian law for marketable wholegrain flours whereas the commercial samples presented in Table 2 had ash values within this limit (apart from three of them which were nevertheless very close to the limit). As for their protein (12.0–13.0% d.m.), lipids (1.8–1.9% d.m.) and total dietary fibre (7.6–12.5% d.m.) content, these commercial flours showed values in general lower than those of samples WGF/SMF in Table 1 (WGF/SMF proteins 12.6–14.1% d.m.; WGF/SMF lipids 1.8–3.0%/1.9–3.0% d.m.; WGF/SMF total dietary fibre 9.8–11.9%/10.2–11.5% d.m.).

As regards total polyphenols we can say that the profile of phenolic compounds in plants depends on their genetics (species and variety) and growing conditions. Phenolic compounds are secondary metabolites that exert a wide variety of functions in plants (reproduction, growth, defence mechanisms, colour and others) [22] and are located accordingly in different plant organs and tissues, especially in the external parts, free or associated with a variety of plant molecules. Soluble (“free”) phenolics are mainly located in cellular vacuoles, whereas insoluble (“bound”) phenolics in cellular walls, bound to other cellular components and especially to fibre, where they contribute to mechanical resistance, growth and morphogenesis, pathogens/stress response and even cellular adhesion [23]. In human nutrition, phenolic compounds are reported to exert a protective action against of a number of human diseases, largely due to their antioxidant properties, whose actual extent and mechanism are still being investigated [22,24,25]. The phenolic compound classes most represented in cereal grains are phenolic acids (especially ferulic) and flavonoids, with the concentration of the single compounds depending on the cereal species. These compounds are present mainly in the cell walls of the aleuronic layer, of the pericarp and of the embryo, much less in the endosperm. Consequently, flours with a higher extraction rate are richer in polyphenols than refined flours [22]. In general, the bound fraction is more abundant than the free fraction (80:20 ratio in whole wheat grains). The ratio hydrolysable/free polyphenols ranged in milling samples reported in Table 3 as follows: WGF 2.3–2.7; SMF 2.2–2.9; RF 1.8–2.4; FB 1.9–3.9; CB 3.1–4.5. In the commercial samples bought in stores and reported in Table 4, even if TPF in the majority of samples were lower than those reported in Table 3 for WGF samples, nevertheless the ratio of hydrolysable/free polyphenols was similar and ranged from 2.2 to 2.7.

Alkylresorcinols (AR) are a group of phenolic lipids mainly found in the outer parts of rye and wheat kernels. More than 99% of total AR content is located in an intermediate layer of the caryopsis that includes the hyaline layer, the testa and the inner pericarp (particularly the outer cuticle of testa and inner cuticle of pericarp), whereas no AR are found in the endosperm or in the germ [26]. Alkylresorcinols content greatly varies in cereal products, with those containing rye and wheat bran having the highest content. [27]. For these reasons, the AR content of different rye and wheat milling fractions has even been proposed as a marker of the presence of bran in flours. Moreover, AR are considered potential biomarkers of the intake of whole rye and wheat-based products for epidemiological research and observational studies [28].

The values found in this study confirm that AR are contained in the outer kernel layers and they are absent in the endosperm [26]. Wholemeal flours (both WGF and SMF) contained between 1/4 and 1/7 of the alkylresorcinols found in the coarse bran which was the richest fraction whereas the fine bran contained, in average, slightly more than half (Table 3). Also, commercial whole meal flours (Table 4) showed the same level of AR found in WGF (Table 3).

The data illustrated in the above paragraphs don’t show significant differences in composition between WGF samples and SMF samples. In addition, calculations from data reported in Table 1 and Table 3 related to RF, FB and CB fractions show that the composition of a flour, recombined by reuniting the roller-milling fractions from each grain mix, results superimposable with that of WGF and SMF from the same grain sample. A difference between milling methods was found regarding particle size distribution instead, with the stone-milled flours presenting a more uniform distribution (all diameters similarly represented) than roller milled reconstituted flours. This is illustrated by Figure 1, showing the average (on the eight grain pools) particle size distribution of flours ground following the two milling methods. It is likely that a different particle size distribution has an impact on the technological quality of flours by affecting water absorption, susceptibility to enzyme attack, interaction with other ingredients and on their nutritional value by affecting nutrients digestibility and bioavailability [2].

Currently, the main Italian law requirement for marketable wholegrain flours (and similar laws apply in other European countries) is that they must have ash between 1.30% and 1.70% d.m. We can say that our seven samples bought on the market were within these limits, whereas none of the stone milled flours from the commercial mills were, all of them being well above the limit. Stone milled flours were given to us by commercial stone mills without indications about possible additional treatments (e.g., sieving) that they might undergo before being marketed. Interestingly, the seven market-purchased flour samples, labelled as “wholegrain” and respecting the law requirements, actually had a composition compatible with the presence of substantially all kernel components, meaning that a slight decortication or sifting could be sufficient to correct the regulated parameter and preserve the nutritional value at the same time.

We must consider that a legal upper limit for ash in flours commercialized as “wholegrain” was set as a safety measure to protect consumers since the grain surface is the most susceptible part to contamination by different agents (contaminants, microorganisms, etc.), in a time when awareness of the nutritional importance of the aleurone and bran had not arose yet and, moreover, white flour was considered superior by consumers for its technological properties, reflected in sensory properties of products. Since, at present, grain cleaning technologies (e.g., brushing and decortication, to improve safety) and bran processing technologies (e.g., micronization, to improve the technological performance of the dough made from wholegrain flour and the organoleptic properties of the products) have evolved, it is possible to find milling procedures that respond to all needs, safety, nutritional and technological/sensory quality. Consequently, the old limits might be changed to reflect the composition of modern wholegrain products.

We are aware that we analyzed a small number of samples although representative of what can be found on the real market, so it is difficult to suggest limits for both traditional and new quality parameters. However, from this study emerges, that total dietary fibre, total lipids and alkylresorcinols can be taken into consideration as possible markers of the presence of the bran fractions and germ in the flours.

## 5. Conclusions

Wholegrain soft wheat flours are currently produced using either stone or roller milling. We were able to demonstrate that stone milling produces a wholemeal flour where all kernel components are present, so it is justified to consider this kind of flour good for consumers’ health. We also demonstrated that in roller milling if the separation of grain constituents is only temporary and they are later recombined in the same proportions as in the original grains, there is no compositional difference between a stone milled or a roller milled flour. We must also observe that roller milling gives the possibility to obtain different fractions which are interesting to produce different functional products if used on their own.

Flour sifting after milling or recombination in proportions different from those in the original bran to comply with current legislation in some countries like Italy produce a flour which has a lower content of some bioactive components with respect to a real wholegrain product, so it is less interesting from a nutritional point of view. The safety of wholegrain flours should be achieved by improving the cleaning steps before grain milling or by applying a minimal peeling if precious components have to kept.

Even if the composition in a stone milled or a roller milled wholegrain flour is the same, at least for the considered parameters, i.e., proteins, lipids, ash, total dietary fibre, total polyphenols and alkylresorcinols, the two technologies produce flours with different particle size distributions. This might have an impact not only on the technological quality of flours, but also on their nutritional quality: the different effects of wholegrain flours on the bioavailability of nutrients would need further and specific studies.

As regards new quality parameters to be used as markers of nutritional quality for wholegrain soft wheat flours, besides proteins and ash content, we studied lipids, total dietary fibre, total polyphenols, and alkylresorcinols. Of course, if limits have to be set in legislation, more results on more samples should be collected to explore the whole range of variability. Further parameters such as damaged starch, phytic acid and wheat germ agglutinin are also going to be studied in our lab.

## Figures and Tables

**Table 1:** Proximate composition of milling products obtained from eight soft wheat grains.

Grain Code	Test Weight (kg/hL)	Milling Product	Extraction Rate (%)	Moisture (%)	Proteins (% d.m.)	CV (%)	Ash (% d.m.)	CV (%)	Lipids (% d.m.)	CV (%)	Total DF (% d.m.)	CV (%)
1	74.1	WGF	100.0	13.9	14.1	0.3	1.91	0.28	3.0	0.6	10.7	3.3
		SMF	100.0	13.1	14.1	0.3	1.90	0.34	3.0	1.6	10.9	0.8
		RF	62.4	14.7	13.1	0.2	0.54	2.38	1.5	1.1	1.8	1.4
		FB	11.0	13.3	16.5	0.4	3.46	0.83	5.1	2.5	20.7	8.4
		CB	26.6	12.9	18.3	1.4	6.14	0.36	6.5	3.7	41.8	1.3
2	73.4	WGF	100.0	14.0	13.6	0.3	1.76	0.70	2.8	1.3	11.1	3.0
		SMF	100.0	12.0	13.7	0.4	1.77	0.64	2.9	0.2	10.7	0.0
		RF	67.0	13.8	12.1	0.3	0.56	1.23	1.6	2.4	2.3	0.0
		FB	16.8	10.5	16.7	0.5	3.38	1.33	6.3	0.3	23.7	0.7
		CB	16.2	12.8	18.5	0.2	6.11	0.34	6.3	1.8	39.9	1.2
3	79.0	WGF	100.0	12.9	12.6	0.8	2.03	1.98	2.9	0.1	10.9	3.0
		SMF	100.0	12.4	12.6	1.1	2.08	2.58	2.9	0.7	10.7	0.0
		RF	71.8	14.9	11.4	1.3	0.82	0.69	1.6	0.7	2.4	0.0
		FB	13.3	13.8	15.7	1.2	3.56	1.03	5.2	1.4	24.6	0.7
		CB	14.9	12.6	17.9	0.9	6.73	0.55	6.3	1.5	39.8	1.2
4	70.3	WGF	100.0	14.0	12.8	0.5	2.41	2.76	2.8	0.1	11.6	2.1
		SMF	100.0	11.4	13.1	0.1	1.97	1.50	2.9	0.7	10.4	3.8
		RF	69.0	13.5	11.3	0.1	0.98	2.84	1.7	1.6	2.7	9.0
		FB	14.5	11.8	16.7	2.3	3.70	0.80	5.6	0.6	24.2	0.3
		CB	16.5	11.7	16.8	0.3	6.03	0.79	6.1	1.5	42.9	0.0
5	74.1	WGF	100.0	15.2	13.3	0.7	2.08	3.19	2.9	0.6	11.9	2.8
		SMF	100.0	12.7	13.4	0.3	2.10	1.23	2.9	0.2	11.2	2.2
		RF	70.0	15.1	12.0	0.0	0.83	1.34	1.6	1.8	2.5	4.0
		FB	16.2	12.7	16.8	0.4	3.79	1.88	5.9	1.7	23.4	2.1
		CB	13.8	10.7	17.5	0.2	6.58	0.84	6.5	1.0	43.2	0.9
6	83.8	WGF	100.0	13.4	13.3	2.2	1.76	1.16	2.9	0.8	10.4	0.8
		SMF	100.0	13.1	13.4	0.4	1.93	1.20	3.0	0.3	10.2	2.4
		RF	77.1	13.6	12.3	0.1	0.72	3.37	1.7	0.8	2.4	3.4
		FB	10.7	12.1	16.4	1.0	3.90	2.82	6.9	2.2	23.7	4.1
		CB	12.2	11.5	18.0	0.3	7.23	2.54	7.5	0.5	42.3	0.4
7	84.0	WGF	100.0	13.0	13.4	0.1	1.78	0.93	1.8	2.8	9.8	5.8
		SMF	100.0	12.2	13.2	0.6	1.94	0.84	1.9	4.8	11.5	0.0
		RF	78.7	13.8	12.2	0.7	0.72	2.04	0.8	2.1	2.6	9.4
		FB	10.3	11.2	16.5	0.6	3.88	0.57	4.2	0.7	27.9	0.6
		CB	11.0	11.1	17.5	1.7	6.93	0.28	7.2	0.6	46.5	1.5
8	84.0	WGF	100.0	12.9	13.1	0.6	1.83	2.80	1.8	0.9	10.6	0.0
		SMF	100.0	12.3	13.2	0.5	1.93	4.78	1.9	0.8	10.2	4.0
		RF	76.0	15.3	12.2	0.2	0.83	2.08	0.7	2.0	2.6	0.0
		FB	11.0	12.5	16.0	0.6	4.15	0.66	3.9	0.6	24.5	1.6
		CB	13.0	11.7	17.5	0.6	7.18	0.62	6.9	0.3	45.5	0.7

DF, dietary fibre; d.m. on dry matter; CV, coefficient of variation (two independent determinations for lipids and dietary fibre, three independent determinations for proteins and ash); WGF, wholegrain flour from Bühler MLI 204 laboratory mill; SMF, Stone-milled flour from commercial mills; RF, Refined flour from experimental roller milling; FB, Fine bran from experimental roller milling; CB, Coarse bran from experimental roller milling.

**Table 2:** Specifications and proximate composition parameters of seven wholemeal soft wheat flours of 6 different brands purchased in stores.

Sample Code	Milling Product *	Total Protein * (% d.m.)	Total Lipids * (% d.m.)	Total Dietary Fibre * (% d.m.)	Ash ^§^ (% d.m.)	CV
C1	Wholegrain	12.0	1.9	9.6	1.66	1%
C2	Wholegrain, stone milled flour	12.0	1.9	8.4	1.58	1%
C3	Wholegrain, stone milled flour	12.0	1.9	9.6	1.77	1%
C4	Wholegrain	13.0	1.8	12.5	1.79	1%
C5	Wholegrain, stone milled flour	12.0	1.9	12.0	1.66	0
C6	Wholegrain	12.2	1.9	7.6	1.55	2%
C7	Wholegrain	13.0	1.8	12.5	1.75	2%

d.m., on dry matter; * Information from product label; ^§^ Determined in our laboratory; CV, Coefficient of Variation (three independent determinations); C, Commercial flour purchased in stores.

**Table 3:** Total polyphenols and alkylresorcinols in milling products obtained from eight soft wheat grains.

Grain Code	Milling Product	Polyphenols (mgGAE/100 d.m.)						Alkylresorcinols (mg/100 g d.m.)	CV
		Free	CV	Hydrolisable Bound	CV	Total	CV		
1	WGF	214	3%	586	2%	800	2%	42.5	2%
	SMF	204	5%	610	3%	814	4%	40.8	4%
	RF	184	5%	396	4%	581	4%	<l.o.d.	
	FB	254	4%	872	1%	1126	2%	91.7	0%
	CB	281	5%	1265	1%	1547	2%	183.6	3%
2	WGF	213	5%	539	0%	752	2%	47.5	7%
	SMF	219	1%	535	0%	754	1%	45.9	8%
	RF	145	5%	355	2%	500	3%	<l.o.d.	
	FB	232	4%	819	2%	1051	3%	68.1	5%
	CB	272	1%	1029	2%	1301	1%	234.1	1%
3	WGF	225	3%	527	2%	752	3%	46.4	13%
	SMF	227	5%	560	1%	788	2%	39.3	6%
	RF	185	3%	337	3%	521	3%	<l.o.d.	
	FB	227	2%	764	2%	991	2%	98.6	12%
	CB	302	4%	1091	1%	1394	2%	168.4	1%
4	WGF	208	3%	544	2%	752	2%	27.2	3%
	SMF	220	2%	496	1%	716	1%	35.1	3%
	RF	155	2%	349	3%	504	3%	<l.o.d.	
	FB	220	5%	419	1%	639	2%	71.5	2%
	CB	259	2%	1106	8%	1365	7%	179.0	5%
5	WGF	201	2%	524	2%	725	2%	39.0	2%
	SMF	204	2%	517	2%	721	2%	36.3	9%
	RF	144	5%	341	3%	485	4%	<l.o.d.	
	FB	237	2%	780	3%	1017	3%	78.1	5%
	CB	278	4%	1044	1%	1322	2%	189.3	10%
6	WGF	184	4%	454	2%	638	3%	45.0	19%
	SMF	182	4%	475	2%	656	2%	43.4	11%
	RF	170	8%	345	2%	515	4%	<l.o.d.	
	FB	211	5%	793	3%	1004	3%	100.1	6%
	CB	252	4%	935	3%	1187	3%	251.2	9%
7	WGF	195	3%	478	2%	673	2%	46.0	5%
	SMF	189	4%	492	1%	681	2%	47.0	4%
	RF	148	1%	326	0%	475	1%	<l.o.d.	
	FB	213	12%	839	2%	1052	4%	106.6	10%
	CB	261	2%	955	2%	1216	2%	241.5	8%
8	WGF	197	2%	457	3%	654	2%	32.1	3%
	SMF	211	5%	460	1%	671	2%	46.2	4%
	RF	180	4%	319	2%	499	3%	<l.o.d.	
	FB	228	4%	673	1%	901	2%	95.5	2%
	CB	287	4%	904	3%	1191	3%	215.1	5%

d.m., on dry matter; <l.o.d., below limit of detection; WGF, wholegrain flour from Bühler MLI 204 laboratory mill; SMF, stone-milled flour from commercial mills; RF, refined flour from experimental roller milling; FB, fine bran from experimental roller milling; CB, coarse bran from experimental roller milling; CV, coefficient of variation (three independent determinations).

**Table 4:** Total polyphenols and alkylresorcinols in seven wholemeal soft wheat flours of six different brands purchased in stores.

Sample Code	Milling Product *	Polyphenols (mgGAE/100 d.m.)						Alkylresorcinols (mg/100 g d.m.)	CV
		Free	CV	Hydrolisable Bound	CV	Total	CV		
C1	Wholegrain flour	168	4%	428	1%	597	2%	32.7	5%
C2	Wholegrain, stone milled flour	206	3%	464	11%	669	8%	28.8	4%
C3	Wholegrain, stone milled flour	164	3%	449	2%	613	3%	32.2	9%
C4	Wholegrain	207	6%	471	3%	678	4%	38.1	5%
C5	Wholegrain, stone milled flour	175	4%	445	5%	619	5%	31.3	1%
C6	Wholegrain	183	2%	433	0%	616	1%	27.9	3%
C7	Wholegrain	206	3%	446	2%	652	2%	31.2	5%

d.m., on dry matter; * Information from product label; <l.o.d., below limit of detection; C, commercial flour purchased in stores; CV, coefficient of variation (three independent determinations).
